# Visual sensitivities tuned by heterochronic shifts in opsin gene expression

**DOI:** 10.1186/1741-7007-6-22

**Published:** 2008-05-23

**Authors:** Karen L Carleton, Tyrone C Spady, J Todd Streelman, Michael R Kidd, William N McFarland, Ellis R Loew

**Affiliations:** 1 Department of Biology, University of Maryland, College Park, MD 20742, USA; 2 National Human Genome Research Institute, National Institutes of Health, Bethesda, MD 20892, USA; 3 School of Biology, Georgia Institute of Technology, Atlanta, GA 30332, USA; 4 Section of Integrative Biology, University of Texas at Austin, Austin, TX 78712, USA; 5 Friday Harbor Laboratories, University of Washington, Friday Harbor, WA 98250, USA; 6 Department of Biomedical Sciences, Cornell University, Ithaca, NY 14853, USA

## Abstract

**Background:**

Cichlid fishes have radiated into hundreds of species in the Great Lakes of Africa. Brightly colored males display on leks and vie to be chosen by females as mates. Strong discrimination by females causes differential male mating success, rapid evolution of male color patterns and, possibly, speciation. In addition to differences in color pattern, Lake Malawi cichlids also show some of the largest known shifts in visual sensitivity among closely related species. These shifts result from modulated expression of seven cone opsin genes. However, the mechanisms for this modulated expression are unknown.

**Results:**

In this work, we ask whether these differences might result from changes in developmental patterning of cone opsin genes. To test this, we compared the developmental pattern of cone opsin gene expression of the Nile tilapia, *Oreochromis niloticus*, with that of several cichlid species from Lake Malawi. In tilapia, quantitative polymerase chain reaction showed that opsin gene expression changes dynamically from a larval gene set through a juvenile set to a final adult set. In contrast, Lake Malawi species showed one of two developmental patterns. In some species, the expressed gene set changes slowly, either retaining the larval pattern or progressing only from larval to juvenile gene sets (neoteny). In the other species, the same genes are expressed in both larvae and adults but correspond to the tilapia adult genes (direct development).

**Conclusion:**

Differences in visual sensitivities among species of Lake Malawi cichlids arise through heterochronic shifts relative to the ontogenetic pattern of the tilapia outgroup. Heterochrony has previously been shown to be a powerful mechanism for change in morphological evolution. We found that altering developmental expression patterns is also an important mechanism for altering sensory systems. These resulting sensory shifts will have major impacts on visual communication and could help drive cichlid speciation.

## Background

Cichlids are a group of rapidly diverging fishes from the Great Lakes of Africa. Each lake harbors a separate endemic radiation of hundreds of species, which have evolved during its recent history. Cichlids are well known for their diversity of feeding specializations and associated trophic morphologies, as well as their beautiful array of color patterns. We have found that their visual systems are also highly diverse, with some of the most variable visual pigments known in vertebrates [1,2].

Visual pigments comprise opsin proteins bound to a vitamin A-based chromophore, typically 11-cis retinal. The amino acid sequence of the opsin determines the spectral absorbance of the pigment. Spectral tuning of the pigment occurs through amino acid substitutions at sites directed into the retinal binding pocket [3]. Visual pigment absorbance may be tuned to the environment, particularly in aquatic systems. As water quality or depth varies, visual pigments evolve to better match the surrounding light field [4-7]. Owing to the tight correspondence between opsin sequence and function, opsins are an excellent system for the study of molecular adaptation [8].

Vertebrates have one rod opsin and four classes of cone opsins [3,9]. The cone opsin genes include very short wavelength sensitive (*SWS1*), short wavelength sensitive (*SWS2*), rhodopsin-like (*RH2*) and long wavelength sensitive (*LWS*) classes. Each covers a unique spectral range [10,11]. Most fishes have opsin genes from each of these opsin classes and can, therefore, produce visual pigments covering a broad spectral range [12-18].

Cichlid fishes possess members of all four cone opsin classes. Additional gene duplications in several of the classes have produced seven distinct cone opsin genes (*SWS1*, *SWS2b*, *SWS2a*, *RH2b*, *RH2aβ*, *RH2aα *and *LWS*). Analysis of opsin protein expression in tilapia, *Oreochromis niloticus*, has shown that these seven genes produce visual pigments which are spectrally distinct from each other [19]. The corresponding pigments in the Lake Malawi cichlid *Metriaclima zebra *match their counterparts in tilapia with only small spectral shifts (Table 1, see [1]).

**Table 1:** Comparison of peak wavelength (nanometers) of maximum absorbance for cichlid cone opsin genes expressed in COS cells and reconstituted with 11-cis retinal and visual pigments measured *in situ *by microspectrophotometry

**Gene**	***SWS1***	***SWS2b***	***SWS2a***	***RH2b***	***RH2aβ***	***RH2aα***	***LWS***
*Metriaclima zebra *[1]	368	423		484	519	528	
Tilapia [19]	360	425	456	472	518	528	560
Tilapia MSP (this work)	359 ± 6	427 ± 8	456 ± 5	483 ± 9	529 ± 12		595 ± 22

Microspectrophotometry (MSP) data are given as the average ± 1 standard deviation and data for *RH2a α *and *β *are analyzed as one group.

Although sequence differences cause subtle changes in opsin absorbance, rather drastic differences have been detected in the visual pigments of Lake Malawi cichlids [1,2,20]. Our previous work has shown that this results from differential expression of unique subsets of the seven cone opsin genes [1,21]. For example, *M. zebra *has visual pigment peak absorbances (λ_max_) of 368, 488 and 535 nm, resulting from expression of *SWS1*, *RH2b *and *RH2a *opsin genes (where *RH2a *includes both *RH2aα *and *RH2aβ*). In contrast, *Dimidiochromis compressiceps *has λ_max _of 447, 535 and 565 nm as the result of expression of the *SWS2a*, *RH2a *and *LWS *opsin genes. We have also shown that tilapia, an outgroup riverine cichlid, expresses each of the opsin genes during some developmental stage. The genes utilized by lake cichlids are, therefore, subsets of the full opsin set retained by tilapia [19]. The mechanism driving the gene expression changes is not known.

**Figure 1:**
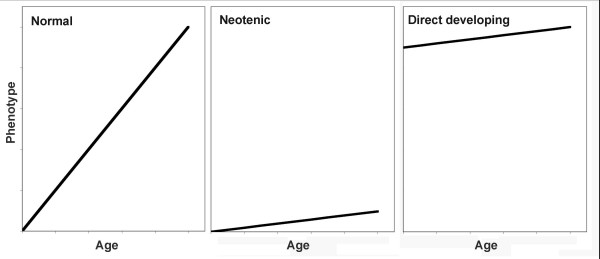
**Impact of heterochronic changes in the rate of change or initial starting conditions of a developmental program on the ontogenetic progression of a phenotypic trait**. In comparison with normal trait development (left), neotenic development shows a reduced rate of change (middle). Direct development (right) shows the adult condition at an early stage with little change in trait value through time.

In this work, we examine whether differential expression results from temporal changes in the developmental sequence of opsin expression. Heterochronic change in developmental patterning is a central idea linking evolution and development. Phenotypic diversity resulting from heterochrony has been demonstrated elegantly for morphological characters [22-26]. A textbook example is salamanders of the genus *Ambystoma*. Here, a decoupling of the developmental programs controlling body development and gonad maturation result in different adult forms [27-30]. In neotenic forms, body development is slowed relative to the gonads such that mature gonads arise within a larval morphology. Plethodontid salamanders have direct developing forms, where the larval phase is abandoned such that only adult morphologies occur throughout development. These ontogenetic programs can be summarized as differences in the rate or timing of different aspects of development (Figure 1).

Although changes in developmental programs are most often associated with morphological differences (size and shape), heterochronic changes have been identified for a variety of traits including bone ossification [31,32], brain differentiation [33], pigment patterning [34] and social behavior [35]. Sensory systems have not yet been examined in terms of heterochrony. However, the close association between visual system sensitivity and opsin gene sequence makes this a natural system to examine the role of developmental timing of opsin gene expression in sensory adaptation.

Studies have shown that visual pigments can differ between larval and adult fishes [13,36-39] including ultraviolet/violet pigment shifts in single cones [40-44] and green/red pigment shifts in double cones [38,41,45]. These ontogenetic shifts could be adaptations to changes in feeding behavior and/or the photic environment following metamorphosis. Shifts can also be more subtle, such as the changes in temporal or spatial expression of *RH2 *or *LWS *gene duplicates that are seen early in the development of zebrafish, *Danio rerio *[14,46-49].

We do not expect cichlid visual pigments to change through ontogeny for two reasons. First, cichlids develop directly without any discrete metamorphosis. The young are mouth-brooded and pass through three developmental stages: embryonic (up to hatching at day 5); larval (from hatching to release from the mother's mouth, day 21); and juvenile (from release to sexual maturity at 6 months) [50,51]. Sexual maturity occurs at the same age in tilapia and the Lake Malawi species. Second, there are no dramatic changes in habitat, water quality or photic environment as the fish develop. For most species, juveniles are released and mature in the same general location where they were spawned. Previous cichlid studies support the developmental stability of the retinal mosaic with no obvious morphological changes. The square retinal mosaic of four double cones surrounding a single cone occurs in *O. niloticus *[52], the Tanganyikan cichlid *Astatotilapia burtoni *[53] and the Lake Victoria species *Haplochromis argens *[54]. The mosaic is formed by day 5 or 6 and remains stable throughout development [53]. However, these studies did not examine opsin gene expression or opsin proteins to test whether visual pigments varied developmentally.

In this work, we used real-time or quantitative reverse-transcription polymerase chain reaction (qRT-PCR) [55-57] to quantify the relative expression of cone opsin genes through ontogeny. We compared the outgroup cichlid, *O. niloticus*, with several species from Lake Malawi. We also used microspectrophotometry (MSP) to confirm that in tilapia, the genes detected by qRT-PCR correspond to visual pigments present in the retina. We were able to accurately quantify expression at multiple time points through ontogeny. This enabled us to map the trajectory of opsin gene expression during the first months of the life cycle and to examine the role of heterochrony in visual sensitivity.

## Results

### Microspectrophotometry

MSP of adult *O. niloticus *revealed three cone morphotypes: single cones with a pigment λ_max _at 449 ± 5 nm and double cones with a pigment λ_max _at either 542 ± 6 nm or 596 ± 6 nm. Tilapia utilize mixed chromophores consisting of retinal derived from both vitamin A1 (11-cis retinal) and vitamin A2 (3,4-didehydroretinal) [58]. Since the most long wavelength sensitive A1-based pigments are 575 nm [10], the presence of a 596 nm pigment is consistent with a mixture of A1 and A2 retinal. Double cone combinations included 542/542 (*n *= 3), 542/596 (*n *= 9) and 596/596 (*n *= 7). Rods contained visual pigments with a peak absorbance of 516 ± 2 nm.

MSP of larval and juvenile tilapia revealed a greater diversity of cone types. These included visual pigments from six distinct spectral classes from the ultraviolet to the LWS pigments (sample spectra shown in Addition file 1). Most individuals had retina utilizing A1 chromophores, although A1/A2 content differed between individuals resulting in variation in λ_max _for a given cone type. As A1/A2 shifts increase with wavelength, shifts were most significant for the LWS pigments. The distribution of cone λ_max _did fall into groups, which were centered around the λ_max _measured from protein expression of the tilapia cone opsin genes reconstituted with the A1 chromophore [19]. Additional file 2 shows the distribution of cone λ_max _as well as the λ_max _determined from the A1 reconstituted proteins and the expected shifts between A1 and A2 pigments [59]. We used the expected A1/A2 sensitivities as well as the observed groupings to sort the pigments into the following gene classes: *SWS1 *(<380 nm), *SWS2b *(410–440 nm), *SWS2a *(445–465 nm), *RH2b *(465–495 nm), *RH2a *(500–545 nm) and *LWS *(>550 nm). Based on this sorting, we calculated the average peak λ_max _± 1 standard deviation (SD) for each class and found they agreed with those determined from protein expression and reconstitution with 11-cis retinal (Table 1) [1,19], allowing for some A1–A2 shift in the *LWS *class. We grouped the *RH2a *α and β cones into one *RH2a *class as there was too much overlap to separate them using MSP.

**Figure 2:**
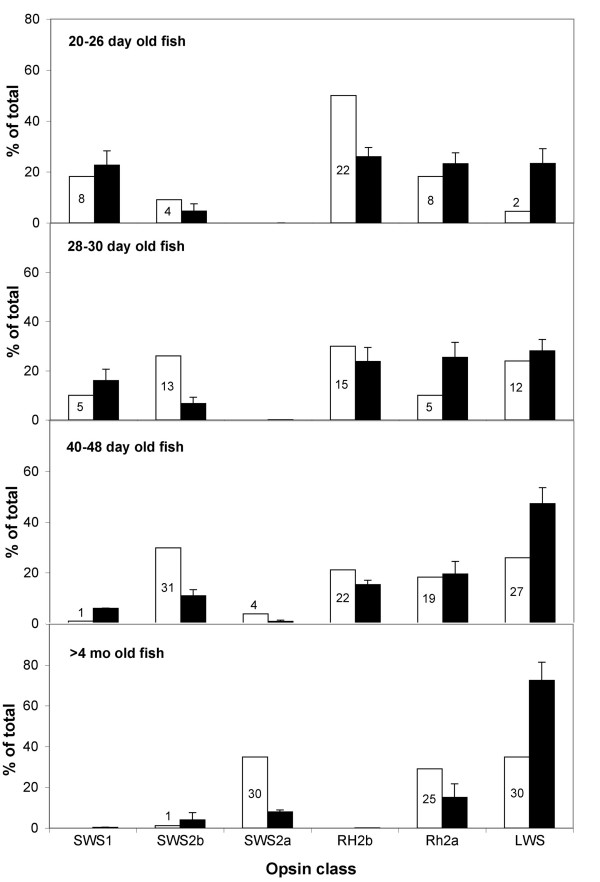
**Cone opsin gene distributions in tilapia, *Oreochromis niloticus*, inferred from microspectrophotometric cone distributions (white bars) or quantified by quantitative reverse transcription polymerase chain reaction (black bars)**. The number of cones quantified by microspectrophotometry is labeled for each white bar. These came from 15 different individuals. Gene expression was averaged over several individuals at each stage: 20–26 dpf (*n *= 8), 28–30 dpf (*n *= 5), 40–48 dpf (*n *= 2), >4 months (*n *= 4).

MSP measurements demonstrated the diversity of cone types present in the retina. Based on the assignment of cone λ_max _to opsin gene, we calculated the relative amounts of opsin expression for each developmental stage (Figure 2). These calculations assume that each cone type expresses only one opsin gene. These relative gene ratios were compared with those determined by qRT-PCR for whole retinas (see below). The ratios were in qualitative agreement and support the idea that when a given gene is transcribed, the corresponding protein is present in the retina.

### Cone opsin expression through tilapia ontogeny

Tilapia opsin expression is turned on by 5 days post-fertilization. This was determined by measuring *RH2a *and *LWS *opsin relative to *β-actin *expression in whole tilapia embryos. The *LWS *opsin to *β-actin *ratio increased from 0.0004 to 0.013 to 0.39 on days 3, 4 and 5. The average *LWS *opsin/*β-actin *ratio during the first 20 days was 0.88 so that day 5 expression was 44% of the larval level. The *RH2a *genes showed similar temporal results. Opsin expression by day 5 is consistent with observations of complete cone outer segments by day 5 in *A. burtoni *[53].

**Figure 3:**
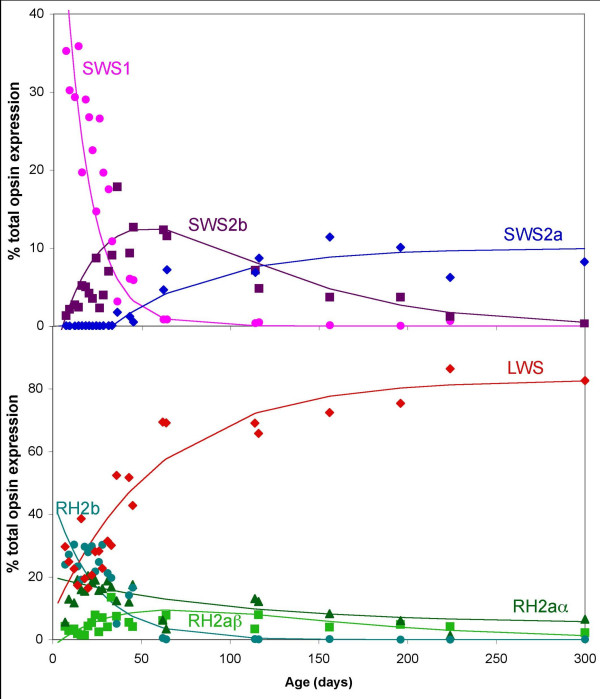
**Relative cone opsin gene expression for tilapia given as a percentage of the total cone opsin expression as a function of age in days (post-fertilization)**. The upper panel shows the expression of shorter wavelength sensitive opsins, which occur in single cones (*SWS1 *●, *SWS2b * and *SWS2a *◆). The lower panel shows the longer wavelength sensitive opsins, which occur in double cones (*RH2b *● *RH2aα *, *RH2aβ *▲ and *LWS *◆). This includes 24 separate time points with three to seven individuals sampled at ages <100 days and one or two individuals for ages >100 days. See Additional file 3 for curves fit to data.

The dynamic changes in cone opsin gene expression through tilapia development are shown in Figure 3. The results from two tilapia broods agreed quite well and are superimposed. Curves have been fitted to the data to summarize the gene expression trends (equations are provided in Additional file 3). The *SWS1 *and *RH2b *genes are expressed strongly in the larval phase, but are not expressed in adults. Expression of both genes steadily decreases until day 60, when they are effectively turned off. The *SWS2b *gene is weakly expressed at the earliest times, peaks in expression around day 60, and then decreases until it is turned off in adulthood. Expression of the *SWS2a *gene does not turn on until day 36, after which it increases steadily until the adult level is reached. *RH2a *genes are expressed throughout ontogeny, but at low levels, while *LWS *increases in expression until it levels off after day 60.

Analysis of the ontogenetic expression patterns using analysis of variance (ANOVA) showed that several of the genes are correlated temporally. *SWS1 *and *RH2b *follow similar time courses and are linearly correlated with each other (*r*^2 ^= 0.81). Both genes are inversely correlated with *LWS *(*SWS1 r*^2 ^= 0.79 and *RH2b r*^2 ^= 0.93) and *SWS2a *(*SWS1 r*^2 ^= 0.59 and *RH2b r*^2 ^= 0.78) genes. Finally *LWS *is positively correlated with *SWS2a *(*r*^2 ^= 0.79) and negatively correlated with *RH2aα *(*r*^2 ^= 0.60). These close temporal associations suggest there may be genetic co-regulation of these genes. This may be the result of the genomic organization of these genes in tandem arrays similar to the opsin genes of other fishes [14].

### Comparison of tilapia qRT-PCR gene expression and MSP

Gene expression determined by qRT-PCR was compared with MSP cone count results by averaging gene expression from several individuals at ages comparable with the individuals examined by MSP (Figure 2). The gene expression measured by qRT-PCR and inferred from MSP cone counts agreed qualitatively at each of the four developmental stages. Whenever a given gene is expressed, we detected the expected cone type using MSP. These developmental comparisons confirmed previous results, which showed agreement between gene expression and the presence of corresponding cone types in adults (see [60] and Carleton et al. unpublished). Therefore, qRT-PCR and MSP provide consistent descriptions of which cones are present at a given developmental stage.

We also quantitatively compared the MSP and qRT-PCR results using regression analysis (Additional file 4). In general, when a given gene's expression increases, more cones are detected. However, the *RH2a *gene showed the opposite trend, suggesting the qRT-PCR and MSP results were not correlated perfectly. There are several reasons why these two methods might differ quantitatively. First, the fish examined by MSP and qRT-PCR came from different broods raised at different times. Second, MSP is not a comprehensive method and inherently involves sub-sampling just a few cones from one or two parts of the retina. Third, MSP convolves gene expression and chromophore usage. This decreases the accuracy of making one-to-one assignments of the cone class to the opsin gene expressed. Fourth, since the photoreceptor outer segments turn over every 2 or 3 weeks [61-63], MSP measures the protein which has been produced during the preceding 2 or 3 weeks. Gene expression measures the genes that are being expressed on a given day. Therefore, the two methods measured proteins or genes across different time frames. For all of these reasons, we use MSP only to make qualitative comparisons and to support the idea that expression of a given gene indicates the presence of the corresponding cone protein at that life stage.

During MSP, cells are identified based on cone morphology (single versus double) providing a link between visual pigment absorbance and cone morphology. We can therefore draw additional conclusions about the progression of gene expression in a given cone type. The 360 (*SWS1*), 425 (*SWS2b*) and 455 nm (*SWS2a*) pigments are all expressed in single cones. Therefore, the qRT-PCR data suggest that there is an ontogenetic progression in single cones, from *SWS1 *to *SWS2b *to *SWS2a*. In double cones, the 480 (*RH2b*), 525 (*RH2a*) and 560 nm (*LWS*) pigments are expressed. Since *RH2a *expression is relatively constant, this suggests that expression of the *RH2b *gene is replaced by *LWS*.

In tilapia, cone opsin gene expression reaches the final adult complement of opsin genes at 225 days, or approximately 6 months (sexual maturity). At this point, the *SWS1*, *SWS2b*, *RH2b *and *RH2aβ *genes are turned off and *SWS2a*, *RH2aα *and *LWS *genes are on. Therefore, the expression of these genes agrees with the observation of three visual pigments in adults by MSP.

### Cone opsin expression through ontogeny of Lake Malawi cichlids

**Figure 4:**
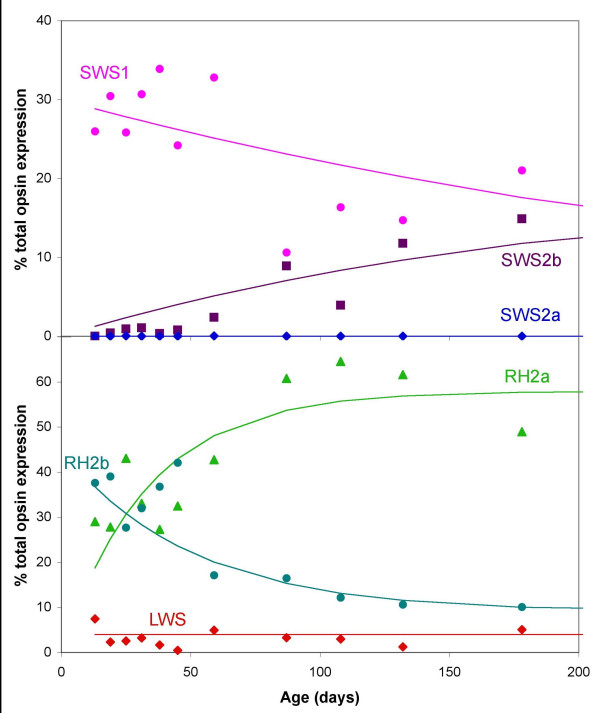
**Relative cone opsin gene expression for *Metriaclima zebra*, given as a percentage of the total cone opsin expression as a function of age in days**. The upper panel shows the expression of shorter wavelength sensitive opsins, which occur in single cones (*SWS1 *●, *SWS2b * and *SWS2a *◆). The lower panel shows the longer wavelength sensitive opsins, which occur in double cones (*RH2b *● *RH2a *▲ and *LWS *◆). The *RH2a *expression is the sum of *RH2aα *and *RH2aβ*. This includes 13 sampled time points with two or three individuals sampled for ages <100 days and one or two individuals for ages >100 days. See Additional file 3 for curves fit to data.

Ontogenetic plots of opsin gene expression in Lake Malawi cichlids showed two different patterns, both of which differ from that seen in tilapia. Species from the rock-dwelling clade (*M. zebra*, *Metriaclima benetos *and *Labeotropheus fuelleborni*) show one ontogenetic pattern (see Figure 4 for data from *M. zebra *and Additional file 5 for data for *M. benetos *and *L. fuelleborni*). For single cone genes, the *SWS1 *gene is turned on in larvae and although it decreases somewhat, it is never turned off. The *SWS2b *gene turns on slowly through development and then stays on. No expression of the *SWS2a *gene is observed. Although *SWS2a *could be expressed under some other behavioral or reproductive state, it is possible that *SWS2a *may now be a pseudogene in these species. We have found missense mutations in *SWS2a *showing that it is a pseudogene in the Tanganyikan cichlid, *Neolamprologus brichardi *[64]. In double cones, the *RH2b *gene is turned on and, although it decreases, it stays on into the adult stage, following a pattern similar to *SWS1*. The *RH2a *genes (*α *and *β *analyzed together) are highly expressed while expression of *LWS *is quite low. These rock-dwelling species, therefore, mimic the expression patterns of young tilapia. They follow the gene progression pattern up through about 40 days post-fertilization (dpf) in tilapia, but stretch this progression out over their life cycle. The Malawian rock-dwelling cichlids have altered their developmental pattern, slowing down the progression of opsin gene expression. Another significant change is that the *RH2a *and *LWS *genes have switched roles. In tilapia, *LWS *is highly expressed and *RH2a *is expressed at a low level. In the Malawian rock dwellers, *RH2a *is highly expressed while *LWS *is expressed at a low level.

**Figure 5:**
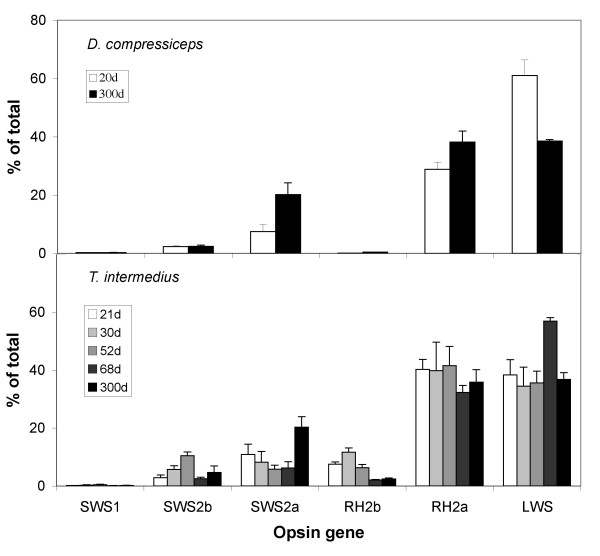
**Relative cone opsin gene expression for several developmental stages in sand-dwelling Lake Malawi cichlids species**. (a) *Dimidiochromis compressiceps*, 20 dpf (*n *= 3) versus adults (*n *= 2); (b) *Tramitichromis intermedius *shown at 21, 30, 52, 68 dpf and adults (*n *= 1 or 2 for each time).

Species from the sand-dwelling clade (*D. compressiceps *and *Tramitichromis intermedius*) show a different ontogenetic pattern of opsin expression (Figure 5). In *D. compressiceps*, 20 dpf fry express the same gene complement as adults, with significant expression of only *SWS2a*, *RH2a *and *LWS*. *T. intermedius *also shows strong similarities between its larval, juvenile and adult expression patterns, which center on *SWS2a*, *RH2a *and *LWS *expression. Therefore, in both sand dwellers, the larval retina appears to have a pattern of gene expression similar to that in adults.

In order to put the different gene expression patterns on a biological scale, which can be plotted through development, we converted gene expression into average peak wavelength of either single or double cones. This was based on a weighting of the relative expression of each gene and its peak spectral absorbance, determined from protein expression (Table 1). For single cones, the *SWS1*, *SWS2b *and *SWS2a *genes are expressed so that the single cone λ_max _is given by

$$\lambda_{\max,S}=\frac{f_{SWS1}\lambda_{SWS1}+f_{SWS2b}\lambda_{SWS2b}+f_{SWS2a}\lambda_{SWS2a}}{f_{SWS1}+f_{SWS2b}+f_{SWS2a}}$$

For double cones, *RH2b*, *RH2a *and *LWS *genes are expressed so that

$$\lambda_{\max,D}=\frac{f_{RH2b}\lambda_{RH2b}+f_{RH2a}\lambda_{RH2a}+f_{LWS}\lambda_{LWS}}{f_{RH2b}+f_{RH2a}+f_{LWS}}$$

**Figure 6:**
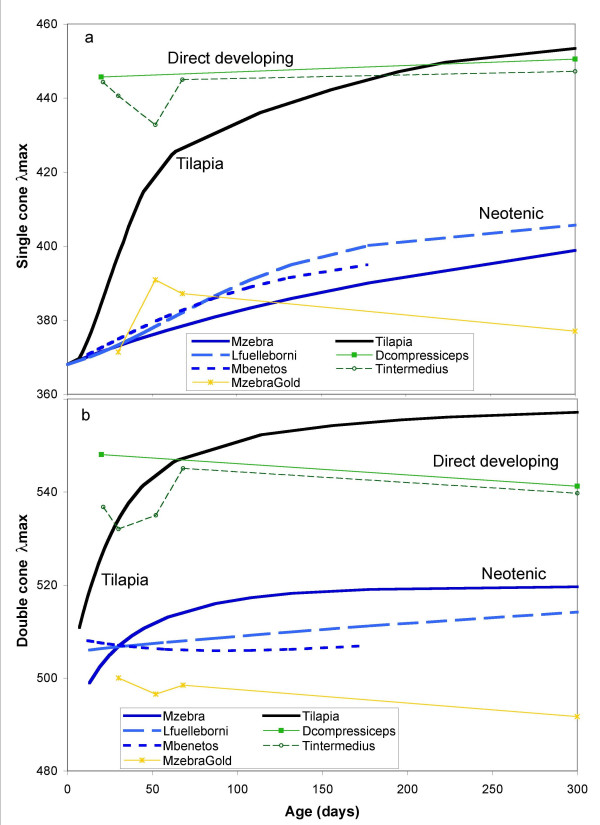
**Spectral peak absorbance (λ_max_) of single and double cones determined by weighting the peak absorbance of each gene by its relative expression level at each developmental stage**. (a) Single and (b) double cones. Curves shown for tilapia (*Oreochromis niloticus*, Figure 3), *Metriaclima zebra *(Figure 4), *M. benetos *(*n *= 11), and *Labeotropheus fuelleborni *(*n *= 13) are taken from least squares fits to developmental data. Points for *Dimidiochromis compressiceps, Tramitichromis intermedius*, and *M. zebra *'gold' are averages for each developmental age.

Here, *f*_*i *_is the fraction of the *i*th opsin expressed out of the total and λ_*i *_is its corresponding peak absorbance. In Figure 6, we plot this average λ_max _for each cone type, as a function of age, to highlight the wavelength shifts that occur through development. We have simplified the data presentation in order to summarize all of the species on one plot and highlight the trends in the data. For those species where we have a significant number of data points (tilapia, *M. benetos*, *M. zebra*, *L. fuelleborni*), we fitted the raw data and plot only the fit curves for clarity (for example, fits from Figures 3 and 4, and Additional file 5; see Additional file 3 for curve fits). For those species where we had fewer data points, we included the raw data in the figure.

The data in Figure 6 map out how cone λ_max _varies through ontogeny. For tilapia, opsin gene expression (Figure 3) shows that single cones progress from *SWS1 *(λ_SWS1 _= 360 nm) to *SWS2b *(λ_SWS2b _= 425 nm) to *SWS2a *(λ_SWS2a _= 456 nm). This gives an average λ_max _for tilapia single cones (black line in Figure 6a) that starts at 360 nm and increases through 425 nm at 60 dpf and ends at 456 nm in adults (300 dpf). For tilapia double cones, gene expression goes from a mixture of *RH2b *and *RH2aα *in early development to *RH2aα *and *LWS *in adults. In Figure 6b, the average λ_max _starts at the average of these two double cone pigments (λ_RH2b _= 472 nm and λ_RH2a _= 528 nm) and ends with double cones with λ_max _close to that of the *LWS *cones (λ_LWS _= 561 nm). Tilapia, therefore, shows an obvious ontogenetic progression from low to high λ_max _in both single and double cones.

The tilapia ontogenetic pattern differs significantly from what we see for the Lake Malawi cichlids. *M. zebra*, *M. zebra *'gold', *M. benetos *and *L. fuelleborni *show λ_max _values that start low in larvae and stay low or rise more slowly than the tilapia progression. Therefore, they retain the characteristics of tilapia larval/juvenile gene expression patterns and can be considered neotenic, with decelerated rates of opsin progression. Both single and double cone λ_max _for *D. compressiceps *and *T. intermedius *start high early in development and remains high into the adult stage, similar to the λ_max _of tilapia adults. This suggests these species have direct developing retinas, which express only adult-like genes resulting in adult-like peak sensitivities.

## Discussion

Differential gene expression, utilizing subsets of the opsin genes, is a powerful mechanism by which the visual system can be tuned through development. In this work, we used qRT-PCR to document the differential expression of opsin genes during development in several cichlid species. This study is one of the first to map out the trajectory of opsin gene expression and to demonstrate that heterochronic shifts can occur in neural systems.

### The tilapia visual system changes through ontogeny

Adult tilapia have a retina based on three spectral classes of cones (449, 542 and 596 nm). The presence of three spectral classes of cone is similar to that reported for other African cichlids [1,2,60,65,66]. These three cone types are a direct result of the expression of three cone opsin genes in adults: *SWS2a*, *RH2a *and *LWS *genes [21].

Larval and juvenile tilapia express different subsets of the opsins and have more complex visual pigment complements. There are periods when four opsin genes are expressed and a brief period around 45–50 days when six cone opsins are present. This dynamic progression of expressed cone opsin genes starts with the short wavelength sensitive genes, *SWS1 *and *RH2b*, which are then replaced with the longer wavelength sensitive juvenile (*SWS2b*) and adult (*SWS2a, LWS*) genes. Ultraviolet/violet sensitivity occurs in many juvenile fishes, as well as fishes that feed on plankton [37,67,68]. The expression of the ultraviolet (*SWS1*) and then violet (*SWS2b*) sensitive genes in the early life stages of tilapia may, therefore, be important for successful foraging.

Another temporal change is that more of the double cones become long wavelength sensitive as the *LWS *gene becomes the dominant opsin expressed in double cones. The shift toward longer wavelength sensitivity may help tilapia adapt to the typically murky African riverine environment [69]. Riverine cichlids studied previously use vitamin A2 chromophores, a factor which may be correlated with more turbid visual environments and selection for longer wavelength sensitivity [6]. This agrees with our previous observations of increases in *LWS *expression and A2 chromophore use in cichlids from the murky habitats of Lake Victoria [60].

Quantifying the temporal patterns of opsin gene expression reveals a dynamic visual system, which changes through tilapia ontogeny. Previous morphological studies indicate that the cichlid retinal mosaic is not restructured through development [53,54], although additional studies are needed to confirm this. If this is true, then opsin expression in individual photoreceptors must change through time resulting in co-expression of more than one visual pigment in a given cell for brief periods. Co-expression has been documented in certain rodents [70,71] and other larval fishes [37,38,72]. In MSP of tilapia, we did not find evidence for co-expression in single cones. However, MSP of the youngest fish is difficult as the cones are quite small and we may not have had a high enough signal-to-noise ratio to distinguish between two pigments with somewhat overlapping spectra. However, we did find approximately 15 double cones at 30–35 dpf having dual absorbance peaks at 490 and 560 nm, indicating expression of both *RH2b *and *LWS *genes. It was possible to bleach the 560 and 490 nm pigments separately, confirming the presence of two distinct proteins. We therefore suggest a model in which the retinal mosaic and its associated neural wiring remains constant, while the complement of cone opsins shifts from shorter to longer opsins as the fish matures. Future work to localize opsins using *in situ *hybridization is needed to confirm retinal spatial patterning and document the succession of opsin genes, in particular photoreceptor cell types.

### Lake Malawi opsin expression exhibits heterochrony

The Lake Malawi species all have the same seven cone opsin genes possessed by their sister group, tilapia. However, the ancestral progression of gene expression has been modified as they adapt to their unique ecologies. Two different developmental patterns were identified here. Larval Malawi rock dwellers express the short wavelength sensitive genes found in young tilapia fry and either retain this larval gene set (*M. zebra *'gold') or slowly shift to the tilapia's juvenile gene set (*M. zebra, M. benetos, L. fuelleborni*). This neotenic expression pattern enables these species to retain the short wavelength sensitivities found in young tilapia. The Malawian sand dwellers, *D. compressiceps *and *T. intermedius*, have a different pattern. They express the long wavelength sensitive genes, similar to adult tilapia. However, they do not follow the same complex ontogenetic progression from shorter to longer wavelength sensitivities seen in tilapia. Instead, they follow a direct development path and express the long wavelength genes beginning in the larval stages.

These changes in developmental program may be an adaptation to the constantly clear waters of Lake Malawi, which is one of the clearest lakes in the world [69]. Fish that inhabit a constant photic environment throughout their life can use the optimal pigment set for those conditions, and may no longer need developmental shifts in visual sensitivity. These developmental changes may also have been driven by the specialized foraging behaviors of these species. Most rock-dwelling cichlids are opportunistic zooplanktivores. Retaining the larval ultraviolet/violet sensitivities into their adult stages may enable them to feed more efficiently on zooplankton throughout their lives [67,73]. The sand dwellers studied here are not known for zooplanktivory and, therefore, may not need ultraviolet/violet detection capabilities.

### Role of heterochrony in sensory systems

This work documents the differing temporal pattern of opsin gene expression in cichlid fishes. Opsin expression can be dynamic, with a progression of different opsin gene combinations from the larval to adult stages. However, Lake Malawi cichlids have modified the developmental program of their cichlid tilapia-like ancestor. Some express the adult gene sets at larval stages while others retain the larval genes into adulthood. These heterochronic shifts cause the large differences in adult sensitivities observed between closely related species [1,2,21]. Heterochrony is, therefore, a key mechanism by which cichlid visual systems are tuned.

**Figure 7:**
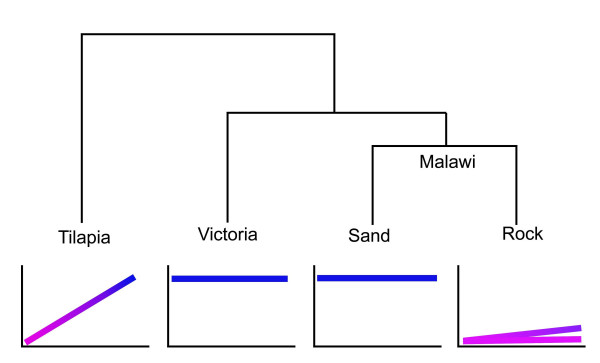
**Developmental programs for opsin expression changes mapped on to the cichlid phylogeny**. Tilapia shows a progression from a larval (pink) to a juvenile (violet) to an adult (blue) gene set. In contrast, cichlids from Lake Victoria and sand dwellers from Lake Malawi have a direct developing retina with only the adult gene set expressed throughout life. Rock dwellers from Lake Malawi have a neotenic progression, retaining the larval gene set or progressing slowly from larval to juvenile gene sets.

These differences have evolved among closely related species which have radiated in Lake Malawi within the last 1 million years. Figure 7 summarizes the cichlid developmental programs within a phylogenetic context including results from Lake Victoria [60]. The rapid evolution of these expression patterns suggests that regulation of the different opsin genes is coordinated by a developmental program that can be easily modified. Studies of additional species are needed to determine how labile these heterochronic shifts are. However, it is clear from this work that evolution can alter early developmental stages to shape adult visual systems. Heterochronic shifts in gene expression are a powerful way to modify the senses.

## Conclusion

Cichlids have diverse visual sensitivities which differ between species. These sensitivities can vary through development, with heterochronic shifts in developmental patterning resulting in alternate adult sensitivities. Changes in adult visual sensitivities could have important implications for mate preferences, mate choice and ultimately cichlid speciation. If closely related species differ in visual sensitivities, females with different visual preferences might prefer differently colored males as mates. Work is in progress to determine whether closely related species differ in visual sensitivities, how these sensitivities relate to the spectral colors of males, and whether these differences affect female mate preferences.

## Methods

### Fish species

Broods of seven different cichlid species were reared in our tropical aquaculture facility according to approved animal care protocols. Nile tilapia, *O. niloticus*, as well as the Malawian species (*D. compressiceps *and *T. intermedius*) were bred from commercial stocks while the Malawian species (*M. zebra, M. zebra *'gold', *M. benetos *and *L. fuelleborni*) were bred from wild caught stocks. Breeding groups were housed in glass tanks and kept at 24–26°C under 14 hour:10 hour day:night light cycles. Light levels were 100 lux at the top of the rack, although this decreased by up to 50% for fish raised on lower levels. The day of breeding was determined when the female's buccal cavity expanded due to a clutch of eggs.

### MSP

Adult tilapia were provided by the Northwest Fisheries Unit of the National Marine Fisheries Service and examined at Friday Harbor. Tilapia at younger developmental stages were raised in our tropical aquaculture facility. A total of 15 individuals were sampled from four different age classes: 20–26 dpf, 28–30 dpf, 40–48 dpf and >4 months in age.

MSP followed previous methods [38]. Fish were dark adapted for at least 2 hours and anesthetized with MS-222. Retinas were dissected under dim red light, placed in buffered saline, teased apart and placed between two quartz coverslips. Spectral absorbance curves from rod and cone cells were obtained using a single-beam microspectrophotometer fitted with quartz optics. Photoreceptor absorbance was measured by scanning in both directions from 750 to 350 nm and comparing with a baseline scan. Pigments were confirmed by their photolability and outer segment dichroism [74]. The λ_max _was estimated by fitting them to a visual pigment template [75]. For each class of photoreceptor cells, the average λ_max _± 1 SD was reported. Photoreceptor cell types were identified by their morphological appearance on the microscope stage.

### qRT-PCR

For tilapia, a preliminary developmental series examined whole embryos from day 2 up to day 25 to look at the onset of *RH2 *and *LWS *opsin expression using *β-actin *for normalization. Full developmental series were then examined for all opsins for two separate tilapia broods (approximately 60 individuals each). Developmental series for *M. zebra, M. benetos *and *L. fuelleborni *were examined from single broods (approximately 30 individuals). Finally, a few developmental time points were sampled for *D. compressiceps*, *M. zebra *'gold' and *T. intermedius*. Either whole eyes (fish <60 days) or retinas (>60 days) were examined for one to three individuals at each time point in the *mbuna *(rock dwellers) and one to seven individuals in tilapia. As cichlid opsin expression has been shown to have a circadian rhythm [76,77], all fish were sampled at the same time of day, between 10 and 11 a.m. Fish were euthanized with MS-222, decapitated and eyes or retinas removed.

The qRT-PCR methods were similar to our previous studies [19]. Total RNA was extracted with Trizol (Invitrogen) and quantified from the ratio of absorption at 260 and 280 nm. One microgram of total RNA was reverse transcribed using a poly T primer and Superscript III (Invitrogen) at 42°C to create a cDNA mixture. Parallel qRT-PCR reactions were then set up using gene-specific Taqman primers and probes [19,21]. The probes were located on an exon-exon junction and no amplification was obtained in the controls without reverse transcriptase enzyme. For the Malawian species, the genetically similar *RH2aα *and *RH2aβ *were analyzed together using primers that amplified both genes, so that qRT-PCR utilized six primer-probe sets (*SWS1*, *SWS2b*, *SWS2a*, *RH2b*, *RH2a *and *LWS*). The same procedure was used for tilapia, except that additional qRT-PCR reactions were performed using forward primers, which distinguished between the *RH2aα *and *β *genes [19]. For the earliest developmental series, *β-actin *was used as a normalization gene with the following primers and probe: forward CCCTGAGGCCCTCTTCCA; reverse GATGCTGTTGTAGGTGGTTTCG; probe 6-FAM-CCTTCCTTGGTATGGAATCCTGCGGA-Tamra. Real-time polymerase chain reaction (PCR) fluorescence was monitored during 40 cycles of PCR on a GeneAmp 5700 sequence detection system (Applied Biosystems, 95°C for 15 seconds, 55°C for 30 seconds, 65°C for 60 seconds). Critical cycle numbers were determined and used to calculate relative gene expression as a fraction of the total cone opsin gene expression as in previous work [19]. Data were collected for two or three replicates at each developmental time point for each developmental series.

## Abbreviations

ANOVA: analysis of variance; dpf: days post-fertilization; MSP: microspectrophotometry; PCR: polymerase chain reaction; qRT-PCR: quantitative reverse transcription polymerase chain reaction; RH: rhodopsin; SD: standard deviation.

## Authors' contributions

KLC, TCS and JTS conceived of the idea for the study. TCS, JTS and MRK generated the fish for the developmental series. WNMcF, KLC and ERL performed and analyzed the microspectrophotometric measurements. KLC and TCS performed the genetic analyses. KLC drafted the manuscript with contributions from all authors. All authors approved the final manuscript (with the exception of WNMcF who approved an earlier version).

## Supplementary Material

Additional file 1 Sample microspectrophotometric spectra for tilapia for all seven cone types: (a)-(c) single cones; (e)-(g) double cones; and (h) rod. These recordings are from a single cell and are representative of the quality of the data. Solid lines are curve fits based on Govardovskii et al. [75] opsin templates. The λ_max _agree reasonably well with those from the expressed opsins of Spady et al. [19] (Table 1), although there are some slight (= 10 nm) differences, which are the result of A1/A2 chromophore shifts in the microspectrophotometry data. Click here for file

Additional file 2 Distribution of λ_max _measured in the tilapia developmental series. The number of cones in each 5 nm bin is indicated. The λ_max _for the A1 reconstituted pigments [19] are marked along the top of the figure as well as the predicted λ_max _of the corresponding A2 pigments calculated using the A1/A2 shifts of Harosi [59]. The boundaries defined by the A1 and A2 pigment λ_max _are shown by the arrows. This suggests that the shorter wavelength pigments are reasonably well resolved, but there are overlaps for the *RH2a *(*α *and *β*) and *LWS *pigments. The assignment of these groups to expression of a particular opsin gene is shown by color coding, with the name of the particular opsin gene similarly colored. We further combine the *RH2aα *and *β *cone pigments into *RH2a *for analysis of opsin gene expression. Click here for file

Additional file 3 The equations used to fit the ontogenetic data for four of the cichlid species examined where we had measured at least 10 time points. In these equations, *y *is the amount of gene expressed and *t *is the developmental age in days. For tilapia, *RH2aα *and *RH2aβ *are measured separately, but for all of the Malawian species, the sum of these two genes is combined as *RH2a*. It is difficult to calculate *R*^2 ^for exponential curves. However, to indicate the quality of the data we calculate *R*^2 ^for the logarithm of the exponential curves (which are linear). Some data involve a rising and then falling part to the curve. *R*^2 ^is labeled by which part of the curve is used. For some of the genes, there is very little expression (for example, *SWS2a *and *LWS *in *mbuna*) or expression is fairly constant over all life stages (*RH2a*). For these genes, the *R*^2 ^values are very low as there is no correlation with age. Click here for file

Additional file 4 Regression analysis used to test the relationships between microspectrophotometric cone numbers and quantitative polymerase chain reaction of gene expression. The regression relationships are noted for each gene along with the *R*^2 ^values. All genes are highly correlated with the caveat that the *RH2a *gene is negatively associated with cone number. See the text for a discussion. Click here for file

Additional file 5Relative cone opsin gene expression for (a) *Metriaclima benetos *and (b) *Labeotropheus fuelleborni *given as a percentage of the total cone opsin expression, as a function of age in days. The upper panel shows the expression of shorter wavelength sensitive opsins, which occur in single cones (*SWS1 *●, *SWS2b * and *SWS2a *◆). The lower panel shows the longer wavelength sensitive opsins, which occur in double cones (*RH2b *●, *RH2a *▲ and *LWS *◆). The *RH2a *expression is the sum of *RH2aα *and *RH2aβ*. Data for *Metriaclima benetos *includes 11 sampled time points with samples of two or three individuals for ages <100 days and one or two individuals for >100 days. See Additional file 3 for curves fit to data.Click here for file
