# Supplementary figures and images for: Visual sensitivities tuned by heterochronic shifts in opsin gene expression
Source: BMC Biol. 2008 May 23;6:22. doi: 10.1186/1741-7007-6-22 (PMC2430543; doi:10.1186/1741-7007-6-22)

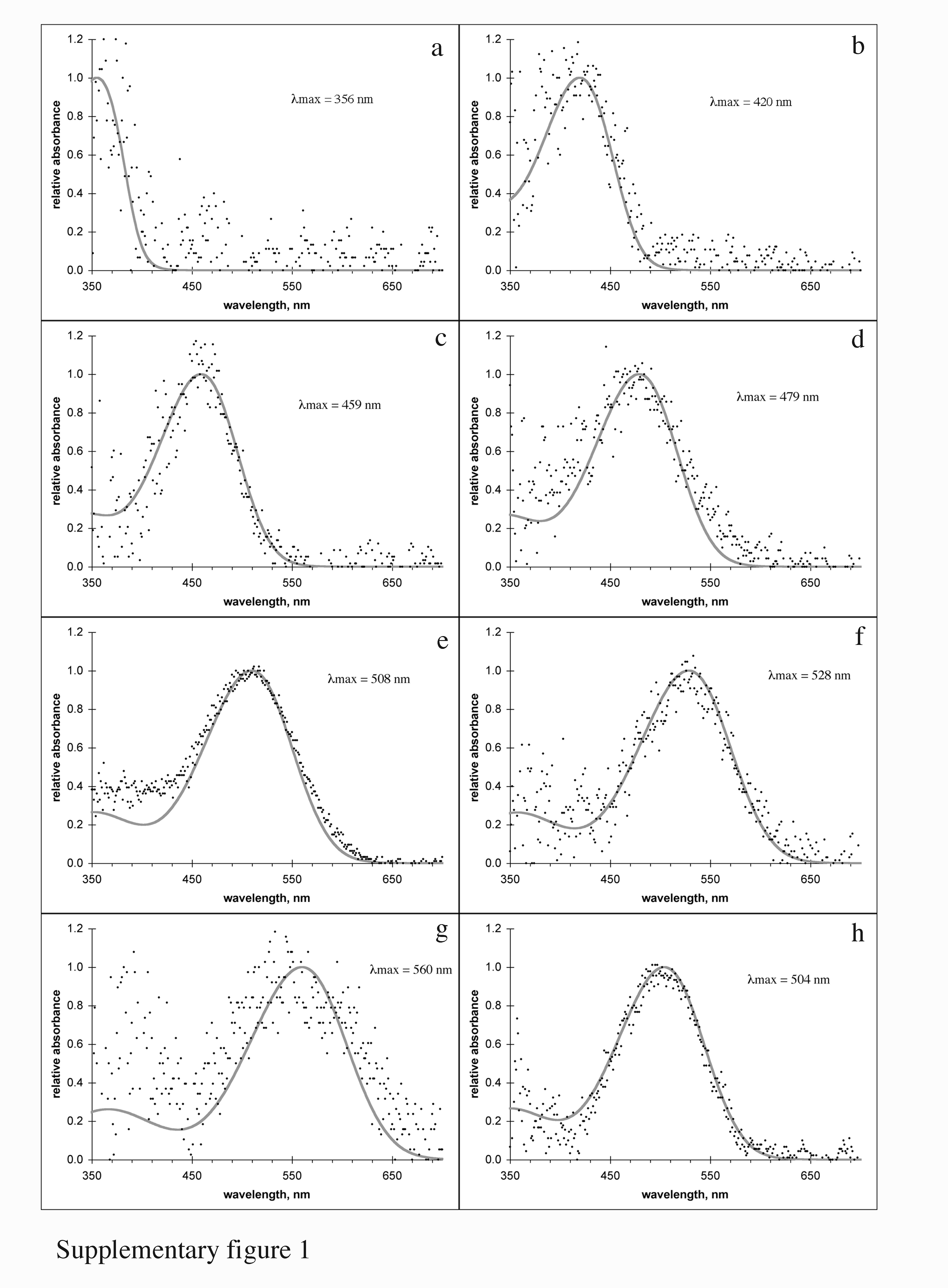

Supplement: Additional file 1 — Sample microspectrophotometric spectra for tilapia for all seven cone types: (a)-(c) single cones; (e)-(g) double cones; and (h) rod. These recordings are from a single cell and are representative of the quality of the data. Solid lines are curve fits based on Govardovskii et al. [75] opsin templates. The λmax agree reasonably well with those from the expressed opsins of Spady et al. [19] (Table 1), although there are some slight (= 10 nm) differences, which are the result of A1/A2 chromophore shifts in the microspectrophotometry data. [file 1741-7007-6-22-S1.jpeg]

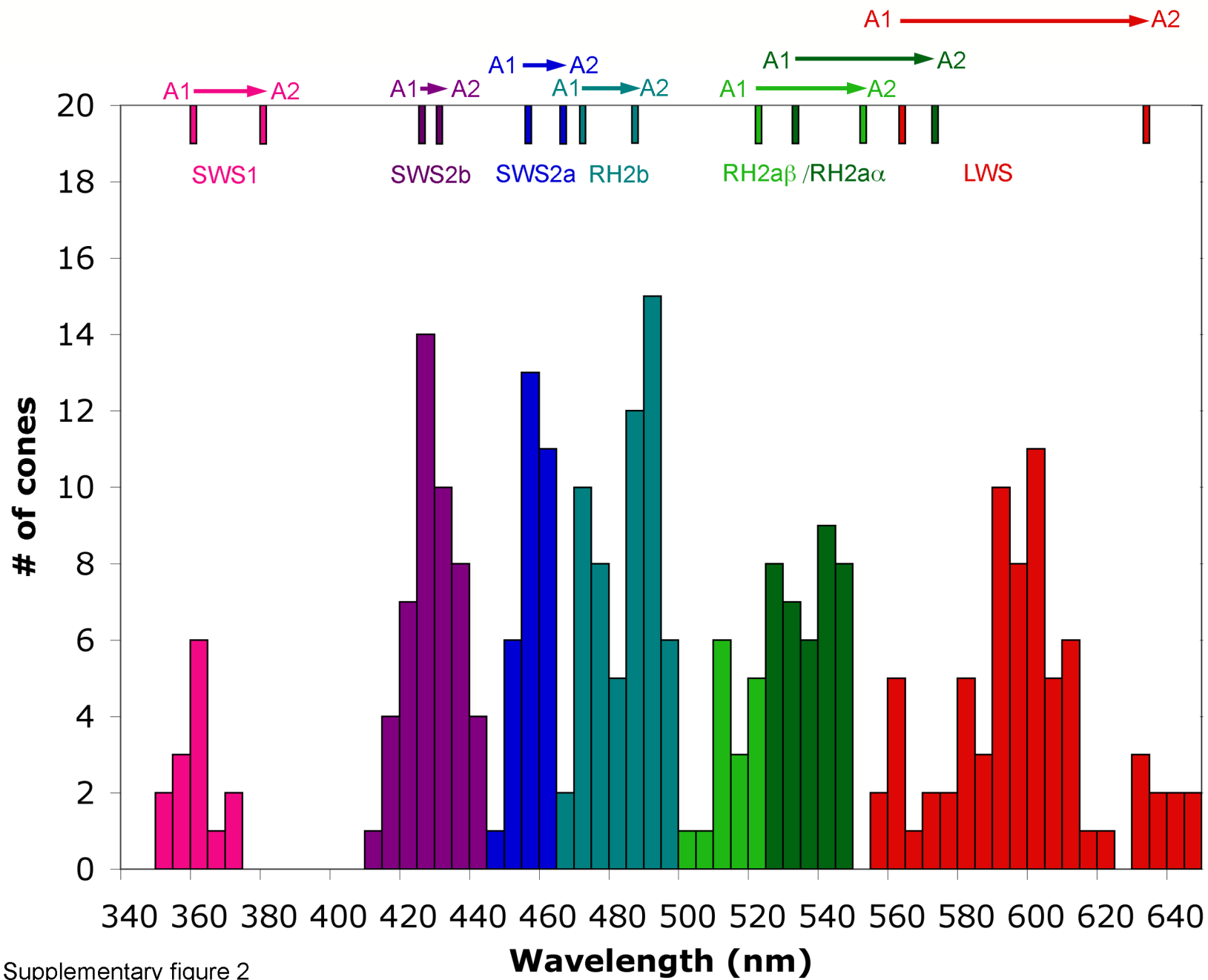

Supplementary figure 2

Supplement: Additional file 2 — Distribution of λmax measured in the tilapia developmental series. The number of cones in each 5 nm bin is indicated. The λmax for the A1 reconstituted pigments [19] are marked along the top of the figure as well as the predicted λmax of the corresponding A2 pigments calculated using the A1/A2 shifts of Harosi [59]. The boundaries defined by the A1 and A2 pigment λmax are shown by the arrows. This suggests that the shorter wavelength pigments are reasonably well resolved, but there are overlaps for the RH2a (α and β) and LWS pigments. The assignment of these groups to expression of a particular opsin gene is shown by color coding, with the name of the particular opsin gene similarly colored. We further combine the RH2aα and β cone pigments into RH2a for analysis of opsin gene expression. [file 1741-7007-6-22-S2.pdf]

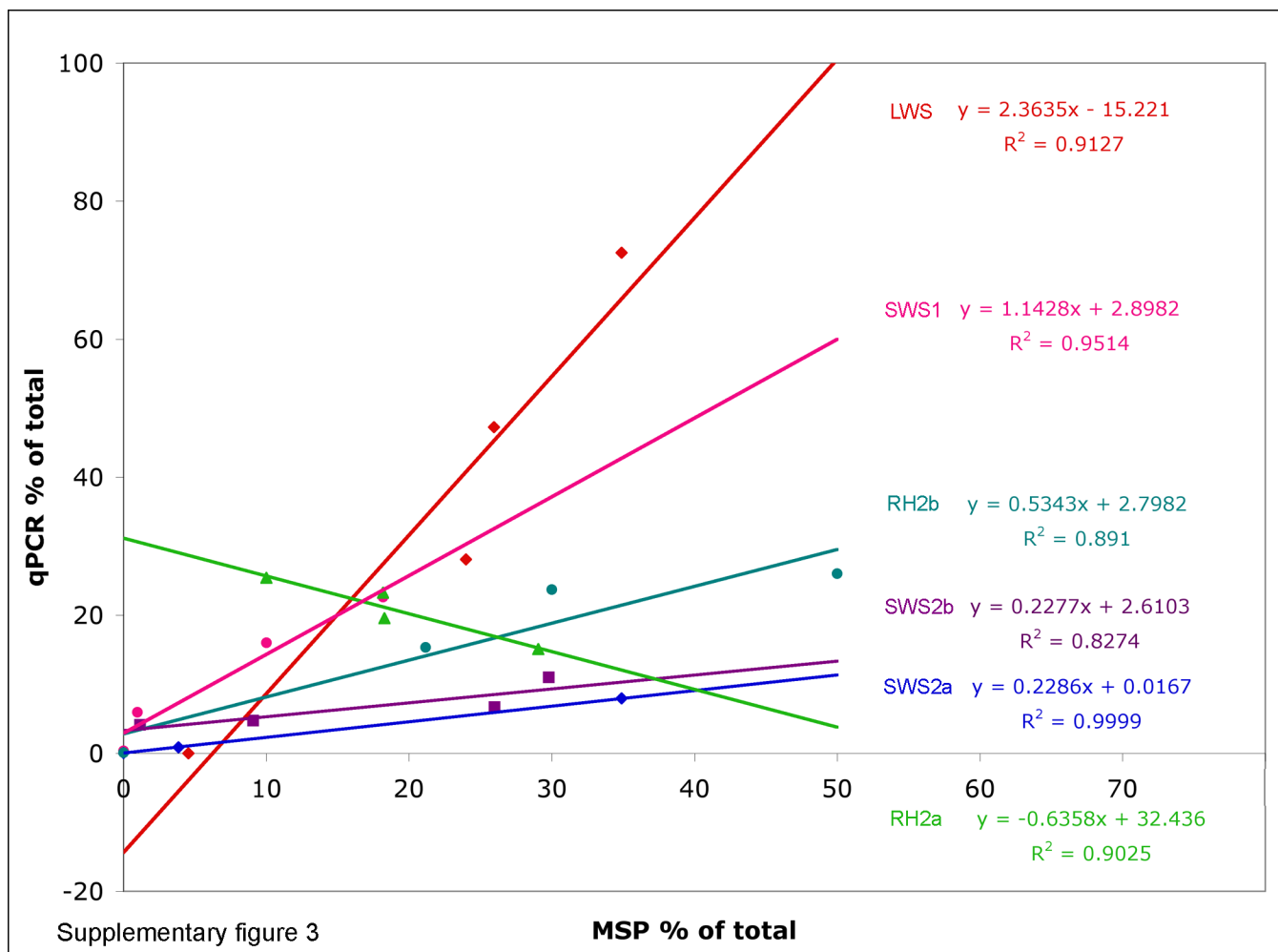

Supplement: Additional file 4 — Regression analysis used to test the relationships between microspectrophotometric cone numbers and quantitative polymerase chain reaction of gene expression. The regression relationships are noted for each gene along with the R2 values. All genes are highly correlated with the caveat that the RH2a gene is negatively associated with cone number. See the text for a discussion. [file 1741-7007-6-22-S4.pdf]

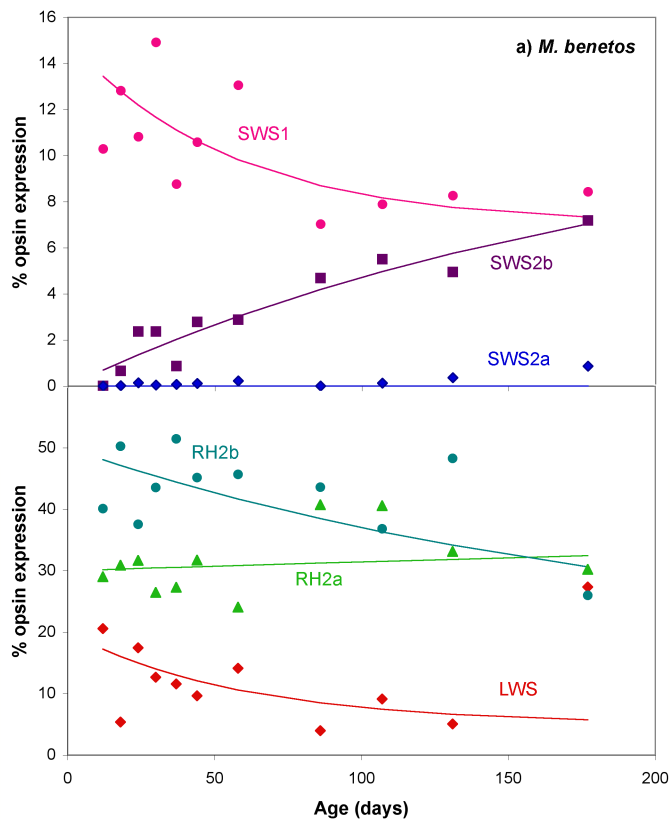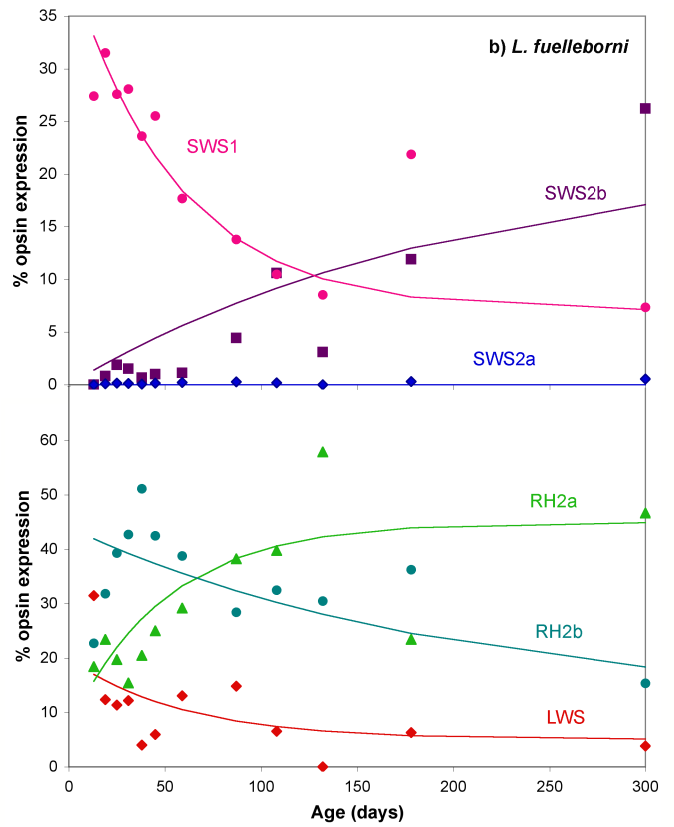

Supplementary figure 4

Supplement: Additional file 5 — Relative cone opsin gene expression for (a) Metriaclima benetos and (b) Labeotropheus fuelleborni given as a percentage of the total cone opsin expression, as a function of age in days. The upper panel shows the expression of shorter wavelength sensitive opsins, which occur in single cones (SWS1 ●, SWS2b and SWS2a ◆). The lower panel shows the longer wavelength sensitive opsins, which occur in double cones (RH2b ●, RH2a ▲ and LWS ◆). The RH2a expression is the sum of RH2aα and RH2aβ. Data for Metriaclima benetos includes 11 sampled time points with samples of two or three individuals for ages <100 days and one or two individuals for >100 days. See Additional file 3 for curves fit to data. [file 1741-7007-6-22-S5.pdf]
