# Supplementary material for: Visual sensitivities tuned by heterochronic shifts in opsin gene expression
Source: BMC Biol. 2008 May 23;6:22. doi: 10.1186/1741-7007-6-22 (PMC2430543; doi:10.1186/1741-7007-6-22)
Supplement: Additional file 3 — The equations used to fit the ontogenetic data for four of the cichlid species examined where we had measured at least 10 time points. In these equations, y is the amount of gene expressed and t is the developmental age in days. For tilapia, RH2aα and RH2aβ are measured separately, but for all of the Malawian species, the sum of these two genes is combined as RH2a. It is difficult to calculate R2 for exponential curves. However, to indicate the quality of the data we calculate R2 for the logarithm of the exponential curves (which are linear). Some data involve a rising and then falling part to the curve. R2 is labeled by which part of the curve is used. For some of the genes, there is very little expression (for example, SWS2a and LWS in mbuna) or expression is fairly constant over all life stages (RH2a). For these genes, the R2 values are very low as there is no correlation with age. [file 1741-7007-6-22-S3.doc]

| **Species** | **Gene** | **Equation** | ***R*^2^ for logarithmic relationship of primary exponential** |
| --- | --- | --- | --- |
| Tilapia | *SWS1* | *y* = 72.4*exp(-0.069*t*) | 0.74 |
|  | *SWS2b* | *y* = 610*[exp(-0.02*t*)-exp(-0.021*t*)] | 0.93 (falling) |
|  | *SWS2a* | *y* 10*[1-exp(-0.0175(*t*-33)] | 0.43 |
|  | *RH2b* | *y* = 45*exp(-0.04*t*) | 0.73 |
|  | *RH2aβ* | *y* = 339*[exp(-0.016*t*)-exp(-0.015*t*)] | 0.37 (falling) |
|  | *RH2aα* | *y* = 5+15*exp(-0.01*t*) | 0.38 |
|  | *LWS* | *y* = 8+75*[1-exp(-0.017*t*)] | 0.91 |
| *Metriaclima zebra* | *SWS1* | *y* = 28.9*exp(-0.003*t*) | 0.50 |
|  | *SWS2b* | *y* = 19.2*[1-exp(-0.005*t*)] | 0.86 |
|  | *SWS2a* | *y* = 0 | 0.006 |
|  | *RH2b* | *y* = 9+36*exp(-0.02*t*) | 0.93 |
|  | *RH2a* | *y* = 58*exp(-0.03*t*) | 0.56 |
|  | *LWS* | *y* = 3.2 | 0.00005 |
| *Metriaclima benetos* | *SWS1* | *y* = 7+8*exp(-0.018*t*) | 0.39 |
|  | *SWS2b* | *y* = 12*[1-exp(-0.005*t*) | 0.92 |
|  | *SWS2a* | *y* = 0.004*t* | 0.68 |
|  | *RH2b* | *y* = 17+33*exp(-0.005*t*) | 0.39 |
|  | *RH2a* | *y* = 30+15*(1-exp(-0.001*t*) | 0.14 |
|  | *LWS* | *y* = 5+15*[exp(-0.017*t*)] | 0.23 |
| *Labeotropheus fuelleborni* | *SWS1* | *y* = 7+33*[exp(-0.018*t*)] | 0.61 |
|  | *SWS2b* | *y* = 22*[1-exp(-0.005*t*)] | 0.68 |
|  | *SWS2a* | *y* = 0.0015*t* | 0.66 |
|  | *RH2b* | *y* = 11+33*[exp(-0.005*t*) | 0.41 |
|  | *RH2a* | *y* = 7+38*[1-exp(-0.02*t*)] | 0.43 |
|  | *LWS* | *y* = 5+15*[exp(-0.017*t*)] | 0.34 |
